# Adhesive Proteins of Stalked and Acorn Barnacles Display Homology with Low Sequence Similarities

**DOI:** 10.1371/journal.pone.0108902

**Published:** 2014-10-08

**Authors:** Jaimie-Leigh Jonker, Florence Abram, Elisabete Pires, Ana Varela Coelho, Ingo Grunwald, Anne Marie Power

**Affiliations:** 1 School of Natural Sciences, National University of Ireland Galway, Galway, Ireland; 2 Instituto de Tecnologia Química e Biológica António Xavier, Universidade Nova de Lisboa, Oeiras, Portugal; 3 Department of Adhesive Bonding and Surfaces, Fraunhofer Institute for Manufacturing Technology and Advanced Materials, Bremen, Germany; Russian Academy of Sciences, Institute for Biological Instrumentation, Russian Federation

## Abstract

Barnacle adhesion underwater is an important phenomenon to understand for the prevention of biofouling and potential biotechnological innovations, yet so far, identifying what makes barnacle glue proteins ‘sticky’ has proved elusive. Examination of a broad range of species within the barnacles may be instructive to identify conserved adhesive domains. We add to extensive information from the acorn barnacles (order Sessilia) by providing the first protein analysis of a stalked barnacle adhesive, *Lepas anatifera* (order Lepadiformes). It was possible to separate the *L. anatifera* adhesive into at least 10 protein bands using SDS-PAGE. Intense bands were present at approximately 30, 70, 90 and 110 kilodaltons (kDa). Mass spectrometry for protein identification was followed by *de novo* sequencing which detected 52 peptides of 7–16 amino acids in length. None of the peptides matched published or unpublished transcriptome sequences, but some amino acid sequence similarity was apparent between *L. anatifera* and closely-related *Dosima fascicularis*. Antibodies against two acorn barnacle proteins (ab-cp-52k and ab-cp-68k) showed cross-reactivity in the adhesive glands of *L. anatifera*. We also analysed the similarity of adhesive proteins across several barnacle taxa, including *Pollicipes pollicipes* (a stalked barnacle in the order Scalpelliformes). Sequence alignment of published expressed sequence tags clearly indicated that *P. pollicipes* possesses homologues for the 19 kDa and 100 kDa proteins in acorn barnacles. Homology aside, sequence similarity in amino acid and gene sequences tended to decline as taxonomic distance increased, with minimum similarities of 18–26%, depending on the gene. The results indicate that some adhesive proteins (e.g. 100 kDa) are more conserved within barnacles than others (20 kDa).

## Introduction

**Figure 1:**
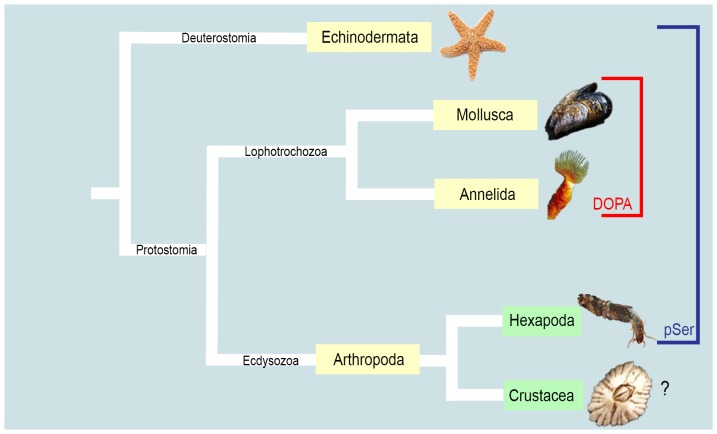
Deep phylogeny, based on molecular data [63], showing animal groups whose adhesion has been examined. Yellow labels indicate taxonomic phyla while green labels indicate subphylum or lower taxonomic rank (in the case of Hexapoda). ‘DOPA’ refers to L-3,4-dihydroxyphenylalanine; ‘pSer’ refers to phosphorylated serines.

Understanding the phenomenon of bioadhesion in wet or humid conditions may greatly aid biotechnological advances in the design of new surgical adhesives or biohybrid and biomimetic materials e.g., [1], [2]. This knowledge will also assist in the prevention of biofouling through design of smarter coatings and surfaces [3]. The metazoan capability of adhering to a substrate, either permanently or temporarily, is particularly pronounced in aquatic invertebrates, as exemplified by the well-studied underwater adhesion of bivalve molluscs, tubeworms, barnacles and echinoderms [4]–[8]. A long history of investigation has shown that similar molecular strategies are used for adhesion by many of these groups, even though they are taxonomically very distinct. Post-translational modifications of protein amino acids (AA) are utilised for adhesion and cohesion; specifically, L-3,4-dihydroxyphenylalanine (DOPA) is present in the adhesive proteins of marine and freshwater molluscs [9], [10] and marine annelids [6]. Similarly, extensive phosphorylated serines (pSer) are present in the adhesive proteins of marine molluscs [11], annelids [6], echinoderms [11] and a freshwater hexapod [12] (Figure 1). DOPA is important for both adhesion and cohesion [13]–[16]. Phosphorylation is less well-studied than DOPA, despite being more widespread across taxa. However phosphate groups on amino acids potentially have both surface adhesive and cohesive functions, as phosphate groups are known to interact strongly with Calcium (Ca^2+^) ions and Ca-containing mineral surfaces [17]. Cross-bridges between phosphate groups may also arise due to Ca^2+^, although this has not been experimentally proven in the context of biological adhesives [12].

Another characteristic of adhesive proteins from both mussels and tubeworms is conserved repeated sequence motifs [18], [19]. The most striking example is mussel foot protein 1 (fp1), which in *Mytilus edulis* contains about 70 repeats of a decapeptide featuring DOPA and hydroxyproline [19]. Homologues of fp-1 have been isolated in a wide range of bivalves in the same taxonomic family (Table 1). While some closely related species have the same or a very similar decapeptide repeated in fp-1, there are also differences between genera. Another major mussel adhesive protein, fp-2, is also characterised by a repeating motif, with 11 repeats of a motif that is also found in the epidermal growth factor-like gene family [20]. Similarly, isolation of some adhesive protein homologues from the tubeworms *Sabellaria alveolata* and *Phragmatopoma californica* show that AA sequences are somewhat conserved [18]. Both species utilised repeating sequences in two adhesive proteins and a third adhesive protein was highly enriched in pSers [18], [21], [22]. In all cases mentioned above, we can observe involvement of a sequence motif, along with one of the post-translational modifications typical in aquatic macro-invertebrate adhesion.

**Table 1:** Repeating amino acid motifs appearing in fp-1 of various mussel species (family Mytilidae), including several species of the genus *Mytilus*.

Species	Amino acid repeat sequence														Reference
*Mytilus edulis*	A	K	P	S	Y	P	P	T	Y	K					[19]
*M. galloprovincialis*	A	K	P	S	Y	P	P	T	Y	K					[58]
*Perna perna* *		K	P	S	Y	P	P	T							[57]
*M. californianus*	P	K	I	S	Y	P	P	T	Y	K					[65]
*M. coruscus*	P	K	I/P	S/T	Y	P	P	T/S	Y	K					[59]
*Choromytilus chorus*	A	K	P	S	Y	P	T	G	Y	K	P	P	V	K	[56]
*P. viridis*	A	T	P	K	P	W	T	A	W	K					[66]
	A	P	P	P	A	W	T	A	W	K					
*P. canaliculus*											P	Y	V	K	[60]
*Aulacomya ater*								A	G	Y	G	G	V	K	[56]
*Trichomya hirsuta*										S	Y	Y	P	K	[60]
*Modiolus modiolus*									S	S	Y	Y	P	K	[60]

*Perna perna fp-1 is an incomplete sequence and is only assumed to be a repeating motif. Underlined residues have been reported to be post-translationally modified.

Alternative adhesive (and cohesive) strategies to the bonding involved in crosslinking DOPA and pSers appear to be found in barnacles as there is evidence that both molecules are absent in the adhesive [23]–[25] or adhesive glands [23]. Unlike mussel adhesive proteins, there has been little evidence in barnacles of repeating sequence motifs. There are currently four barnacle adhesive proteins that have been extensively characterised: cp-100k, cp-52k, cp-20k and cp-19k, although additional proteins are also suggested to be involved [26]. Amino acid composition of cp-19k homologous proteins across barnacle species have reported high levels of serine, threonine, glycine, alanine, valine and lysine [27]. Regular repetition of cysteine (Cys) residues are present in the small adhesive protein cp-20k [28], in the form Cys-Xaa-Xaa-Xaa-Xaa-Xaa-Cys, creating a novel three-dimensional structure supported by disulfide bonds and β-hairpins [29]. This structural motif appears to be conserved across species [30], but interestingly, there is no evidence that Cys in cp-20k participates in inter-molecular disulfide bonding [31]–[33]. The significant hydrophobicity of the two large proteins that make up the bulk of the barnacle adhesive (cp-100k and cp-52k) indicates some involvement of hydrophobic interactions in this adhesive system [33]. The predicted cross-β-sheet secondary structure of cp-100k may play a role in cohesive strength [34]. However, overall no covalent bonding mechanisms have been identified to date in barnacles and clear functional motifs, such as those found in other adhering marine animals, are notably lacking.

Homologues of characterised barnacle adhesive proteins have been reported in multiple acorn barnacle species, however sequence similarity was observed to be low [8], [27] and size and pI of homologous proteins were also inconsistent [27], [30]. A low sequence similarity is common in marine invertebrate adhesives; however barnacles also lack repeating motifs, apart from Cys in cp-20k, which appears more structural in significance. Although some glycosylation of barnacle adhesive proteins is present [33], [35], other post-translational modifications have not been reported in barnacles thus far.

**Figure 2:**
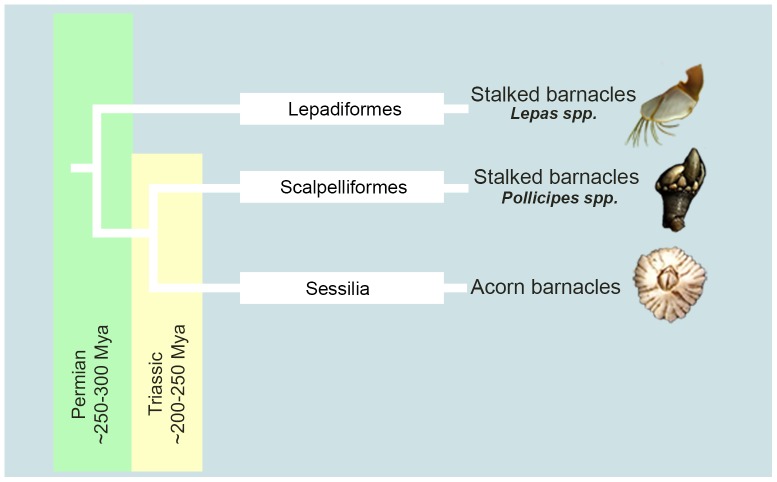
Phylogeny of taxonomic orders of barnacle showing approximate time that these taxonomic groups split, based on [64]. The names of the taxonomic orders of barnacle appear on the tree branches; ‘Mya’ =  million years ago.

The barnacles studied to date are from the taxonomic order Sessilia (acorn barnacles) and therefore extending analyses to stalked barnacles (orders Lepadiformes and Scalpelliformes) greatly expands the taxonomic basis for understanding what is conserved for molecular adhesion in barnacles as a whole (Figure 2). The present investigation is the first published report of *Lepas anatifera* adhesive proteins (order Lepadiformes), and goes into greater depth than a previously published report [36]. This will be accomplished through separation of *L. anatifera* adhesive proteins and *via* data for 52 peptides sequenced *de novo* from mass spectroscopy. Secondly we will use immunohistochemistry to examine cross-reactivity in *L. anatifera* tissue samples using polyclonal antibodies raised against acorn barnacle adhesive proteins. Finally we will analyse and discuss conservation of adhesive domains (using sequence alignment) for different taxonomic orders of barnacle.

## Materials and Methods

### Study organism and adhesive collection

**Figure 3:**
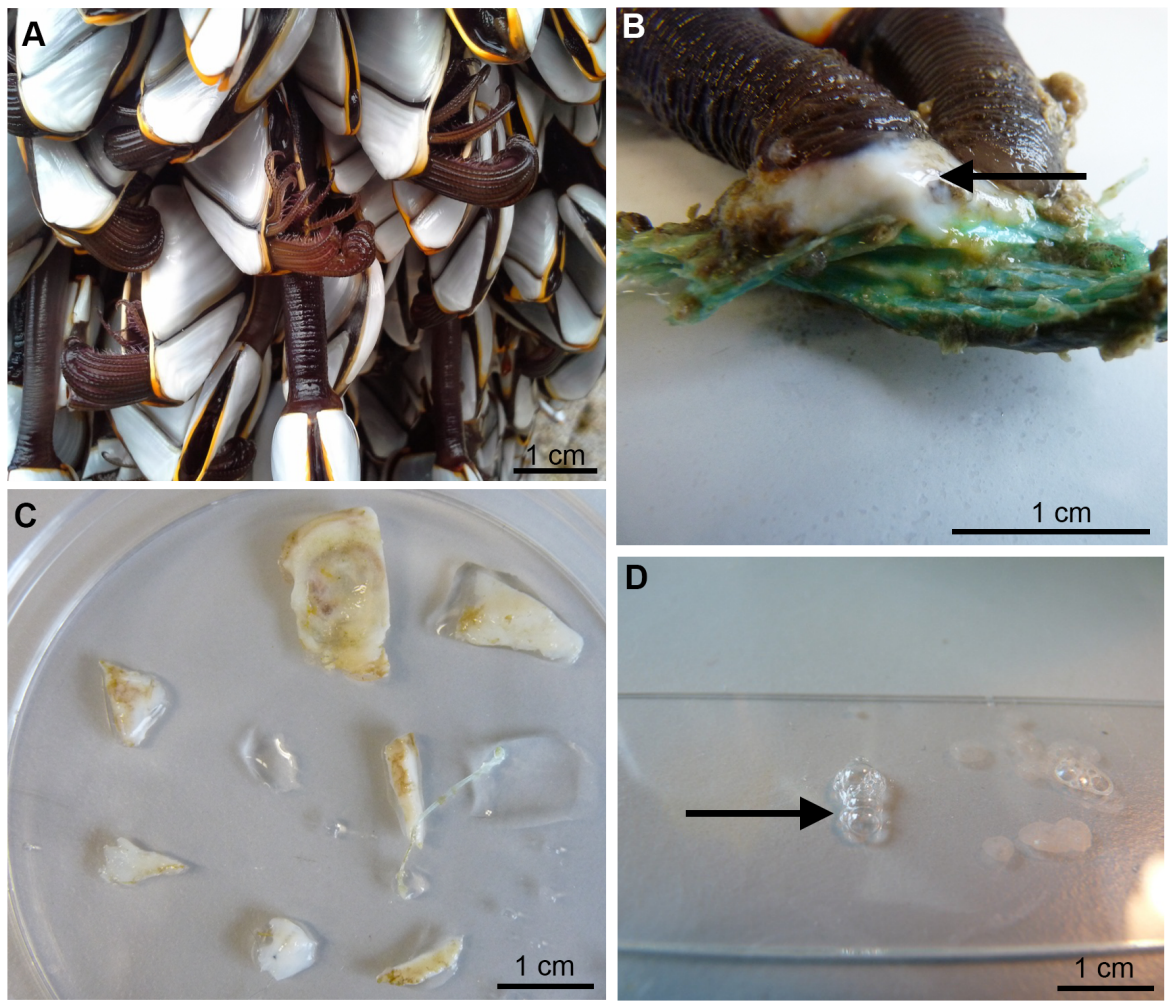
The stalked barnacle *Lepas anatifera* and its adhesive. A) aggregation of *L. anatifera*; B) adhesive plaque of *L. anatifera* (black arrow) adhered to nylon rope; C) samples from the adhesive plaque of *L. anatifera*; D) liquid adhesive extracted from beneath the adhesive plaque of *L. anatifera* (black arrow).

*Lepas anatifera* is a stalked barnacle and a fouling species which attaches to marine installations or debris including plastic, wood or glass, and floats in the ocean across tropical and temperate latitudes [37], [38]. It possesses a membranous base which distinguishes it from many acorn barnacle species that have calcareous bases. The adhesive of *L. anatifera* was collected from samples taken from the wild, which had adhered to various substrates: glass, painted metal (data buoys), plastic and nylon rope (Figure 3). The adhesive extended over the base of the peduncle; it generally had a rubbery consistency and small pieces could easily be pulled away from the cuticle of the barnacle with sterile forceps or sliced from the thick adhesive plaque with a clean razor blade. Great care was taken to not pierce the cuticle of the barnacle, and adhesive from any animal that was wounded was not used for later investigations. The collected adhesive was examined under a stereomicroscope and any visible pieces of dirt, algae and other debris were removed.

### Protein separation and mass spectrometry

Adhesive samples were freeze-dried and solubilised using a urea buffer (7 M urea, 2 M thiourea, 1% (w/v) DTT, 4% (w/v) CHAPS). Freeze-dried adhesive (1.0 mg) was combined with 500 µL buffer, heated to 35°C for one hour, with regular vortexing, then centrifuged for 10 minutes at 20,000 rpm, with the clear supernatant being used for protein separation. SDS-PAGE was performed according to general protocols using a 12% acrylamide gel and a broad range protein molecular mass marker (Promega). Gels were run at 150 V for 30 minutes and stained in Coomassie blue (ThermoScientific). Protein bands of 30, 70, 90, and 110 kD from SDS-PAGE were subjected to trypsin digestion. Briefly, modified trypsin (6.7 ng/µL in 50 mM ammonium bicarbonate, Promega) was added to the dried gel plugs and incubated at 37°C overnight. The obtained supernatant was recovered and gel plugs were further incubated with sufficient volume of 5% (v/v) formic acid and ACN in order to extract higher molecular mass peptides. The recovered supernatant was pooled with the first digest, vacuum-dried and resuspended in 5% (v/v) formic acid. Desalting and concentration of the acidified supernatants containing the tryptic peptides was carried out with chromatographic microcolumns using GELoader tips packed with POROS R2 (20 µm bead size, Applied Biosystems) which were then directly eluted onto the MALDI target plate using 0.5 µl of 5 mg/ml α-CHCA (α-ciano-4-hydroxy-trans-cinnamic acid, Sigma) in 50% (v/v) ACN with 2.5% (v/v) formic acid and air-dried.

Tandem mass spectrometry was performed using a MALDI-TOF/TOF 4800 plus mass spectrometer (Applied Biosystems). Each reflector MS spectrum was collected in a result-independent acquisition mode, typically using 1000 laser shots per spectra and a fixed laser intensity of 3500 V. The fifteen strongest precursors were selected for MS/MS, the weakest precursors being fragmented first. MS/MS analyses were performed using CID (Collision Induced Dissociation) assisted with air, with collision energy of 1 kV and gas pressure of 1×10^−6^ torr. Two thousand laser shots were collected for each MS/MS spectrum using a fixed laser intensity of 4500 V. Searches were performed with MASCOT (version 2.2; Matrix Science, Boston, MA) in the MS/MS ion search mode and the parameters were set as follows: minimum mass accuracy of 30 ppm for the parent ions, an error of 0.3 Da for the fragments, one missed cleavage in peptide masses, and Cys carbamidomethylation and Met oxidation as fixed and variable amino acid modifications, respectively. Peptides were only considered if the ion score indicated extensive homology (p<0.05). In order to extend the protein identifications, searches were also performed with ProteinPilot (Protein Pilot software version 3.0, revision 114732; Applied Biosystems, USA) without taxonomic restrictions and search parameters were set as follows: enzyme, trypsin; Cys alkylation, iodoacetamide; special factor, gel-based ID; and ID focus, biological modification and amino acid substitution.

### Peptide de novo assignment of MS/MS spectra

*De novo* peptide sequencing was conducted with Peaks Studio 5.3 software (Bioinformatics Solutions Inc., Waterloo, ON Canada). At first, a data refinement step was performed with a quality threshold set at 1.0. *De novo* sequencing was then carried out with the following parameters: a parent mass error tolerance of 5.0 ppm, a fragment mass error tolerance of 0.1 Da, cysteine carbamidomethylation as fixed modification, and methionine oxidation and glutamine and asparagine deamidation as variable modification. More stringent criteria were applied with a parent mass error tolerance of 2 ppm with high resolution mode for both MS and MS/MS. Successively, trypsin, semi-trypsin and no-enzyme were chosen as enzyme specificities. Proposed amino acid sequences were then sorted by their Average of Local Confidence (ALC) in order to choose the best spectra to annotate.

### Immunohistochemistry

Polyclonal antibodies against cp-52k, cp-68k and cp-100k from *Megabalanus rosa* were provided by Professor K. Kamino. The acorn barnacle *Amphibalanus improvisus* was used as a positive control, as it has been shown that the adhesive proteins cp-100k and cp-68k are present in *Amphibalanus* species [8]. Paraffin embedded samples of *L. anatifera* and *A. improvisus* (fixed in Bouin's fluid) were sectioned at a thickness of 5 µm, then deparaffinised, rehydrated and moved into TBS (Tris-buffered saline) (pH 7.6). Antigen retrieval was performed by heating to 98°C in a sodium citrate buffer (10 mM sodium citrate, 0.05% (v/v) Tween, pH 6.0) for 20 minutes. Samples were then incubated in 3% (v/v) H_2_O_2_ for 30 minutes and non-specific binding was blocked by incubation in 4% (v/v) normal goat serum for 2 hours (room temperature). Polyclonal antibodies against cp-52k, cp-68k and cp-100k (raised in rabbits) were diluted to 1∶1000 in blocking solution. Primary antibodies were applied to sections and incubated overnight at 4°C. After washing in TBS with 0.025% Triton, the Rabbit ExtraAvidin Peroxidase staining kit (Sigma-Aldrich) was applied according to the manufacturer's directions. Colour was developed with AEC chromogen (Sigma-Aldrich) for 5 minutes, then washed in running tap water for 5 minutes. Negative controls were processed alongside each experiment, without the addition of the primary antibody.

### Sequence alignment

Published sequences for cp-19k and cp-20k adhesive proteins were available for several acorn barnacle species. The only published sequence for cp-100k was from *M. rosa*; this was used in conjunction with unpublished cp-100k sequences for *A. amphitrite* and *Fistulobalanus albicostatus* provided by Professor K. Kamino. For stalked barnacles, an NCBI tBLASTx (http://blast.ncbi.nlm.nih.gov) search for homologues to cp-100k produced EST sequences from *Pollicipes pollicipes* [39] that could be aligned with parts of the cp-100k gene and cp-19k gene. These ESTs were included in the analysis because we could not find any reference to these homologies in the literature. Both nucleotide and amino acid sequences were aligned with MEGA5 [40] using ClustalW alignment. Identity and similarity calculations were obtained using the GeneDoc program [41].

## Results

### Solubilisation and separation

**Figure 4:**
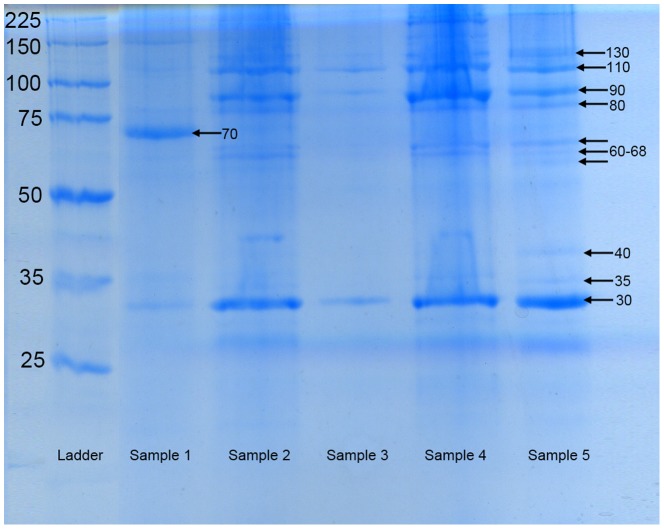
SDS-PAGE adhesive protein separation of five biological replicates (‘samples’) of *L. anatifera*. Left hand column is ladder of protein molecular masses. Prominent, repeated bands were observed at approximately 30 kDa, 90 kDa and 110 kDa. Fainter bands were detected at 35 kDa, 40 kDa, 60–68 kDa, 80 kDa, and 130 kD kDa. A band at 70 kDa was sometimes intense and weak at other times.

The hardened adhesive was almost completely solubilised using a buffer of urea and thiourea with DTT as a reductant. It was possible to separate the supernatant into at least 10 protein bands using SDS-PAGE. Biological replicates were run with *L. anatifera* adhesive samples collected from different sampling dates and locations and the gel separation profile observed was similar each time. Intense bands were present at approximately 30, 70, 90 and 110 kDa, although the band at 70 kDa was not intense on all occasions (Figure 4). The protein separation results from the current study were compared to two investigations of the adhesive proteins of the stalked barnacle *Dosima fascicularis*, which is a close relative of *L. anatifera* [42], [43] (Figure S1). Protein bands at approximately 30, 70, 90 and 110 kDa were consistently observed in both *L. anatifera* and *D. fascicularis* (not including other weaker bands) (Table 2). Bands indicating larger >140 kDa were observed in both species, but these were not analysed in more detail at this stage.

**Table 2:** Protein masses (kDa) of gel bands observed in the polymerised adhesive of *L. anatifera* (current study) and *D. fascicularis*; 1: polymerised adhesive [43], 2: partially polymerised adhesive [42], 3: unpolymerised adhesive [42].

L. anatifera	D. fascicularis		
	1	2	3
26w		24	18
30*			**30**
35w			
40w	47		
60–68w	63		
**70**	**68**	**70**	**70**
80w		75	
90*	**85**	**90**	**85**
110*	**111**		**110**
130w*		140	140

See Figure S1. An error of 5 kDa was considered for this comparison. The most consistently found bands present in both species are indicated in bold, bands that were consistent within *L. anatifera* during repeated SDS-PAGE analyses are indicated with *, bands that were weak in *L. anatifera* adhesive are indicated with ‘w’.

### Analysis of protein bands with mass spectrometry

Protein bands were excised from SDS-PAGE gels and used for identification by mass spectrometry. The spectra produced for each protein band had no confident database matches thus further analysis was conducted using *de novo* sequencing. The trypsin digests of each band were analysed and produced 52 short sequences ranging in molecular mass from 815 to 1770 daltons (7-16 AA in length) (Table 3). Several of the sequences found were repeated across several of the bands that were analysed. For example, the peptides YSPMFSR and MPAKPLPR appeared in 30, 70, 90 and 110 kDa bands. Other peptides appearing in more than one band were YLSSLLFGR (70, 90 kDa), FSQPYFYVPYR (30, 110 kDa) and NYMLFTTR (70, 90, 110 kDa).

**Table 3:** *De novo* sequences from tryptic peptides of *L. anatifera* adhesive protein bands (listed along top row), analysed by PEAKS using MALDI-TOF/TOF MS.

m+H^+^	30	70	90	110
**815.47**			mpallvr	
**855.11**		mslmmsr		
**870.01**			ltpsslpr	
**870.50**	wlvslpr			
**903.39**	yspmfsr	yspmfsr	yspmfsr	yspmfsr
**909.09**				lpgawipr
**909.16**	mpakplpr	mpakplpr	mpakplpr	mpakplpr
**990.03**				saspertsr
**990.08**			rwssngkr	
**990.50**	vgadssgngar			
**1055.54**		ylssllfgr	ylssllfgr	
**1061.50**		nymlfttr	nymlfttr	nymlfttr
**1106.24**				sarylganvr
**1106.55**	snlylqnvr snlsnygpvr			
**1122.24**	gfsrssnlvr			
**1122.59**	ghgsalnlvr			
**1147.49**	yysfpsdlr			
**1158.33**	qmvfyidsr			qmvfyidsr
**1181.64**		ldnglnvhsgr venlvgglkpr	nnnlvgglkpr	
**1247.55**				dmhpffnpsr dmhprhgnqr
**1250.57**	ysghlgflnsr			
**1337.36**		gsgatpysrggdgr		
**1371.64**	anfsplvssffr			
**1393.51**				qgssrfnisknr
**1431.75**		dpmplpvpsllpr		
**1443.70**			fslfnvptlysr	
**1444.71**		dgsreaaylplpr rmkeaaylplpr		
**1445.70**				ypreaataavsgpr
**1459.72**		ypgleaataaqlvr		
**1466.64**	fsqpyfyvpyr			fsqpyfyvpyr
**1500.74**		ypglkpstaanllr ypglqpstaanllr		
**1520.75**	qqlalpsfvsqfr			
**1530.71**				rmpcaataavsgpr
**1674.73**			fedflvsnvqsfsr	
**1770.93**			vvlvakshnslyvegr	

Monoisotopic masses of each tryptic peptide are listed in the first column. Sequences found in more than one protein band are underlined.

No significant homologies were found for any *de novo* sequences in general protein databases (NCBI and UniProt). The longest *de novo* sequences (those which are >11 AA in length) were also subject to multiple BLAST analyses and compared to translated cDNA open reading frame sequences from an unpublished transcriptome database of *Amphibalanus amphitrite* [30], [44] and an unpublished database for *Tetraclita* [45]. However these databases searches returned no matches with *L. anatifera* peptides (Yue Him Wong, pers. comm. August 2013). Extensive efforts to isolate genes corresponding to the *de novo* sequence fragments using RACE PCR were unsuccessful (data not shown).

Peptide sequences from two previous *D. fascicularis* studies were compared with those determined from *L. anatifera* isolated proteins. The *L. anatifera* sequence MPALLVR, found in the 90 kDa protein band, was present as YPALLVR in a 70 kDa protein band from *D. fascicularis* adhesive [42]. Sequence homologies were also observed between the peptides WLVSLPR (*L. anatifera* 30 kDa) and AATVSLPR (*D. fascicularis* 14 kDa [42]), and FEDFLVSNVQSFSR (*L. anatifera* 90 kDa) and FEDFLVNNLNAFSR (*D. fascicularis* 63 kDa [43]).

### Immunohistochemistry

**Figure 5:**
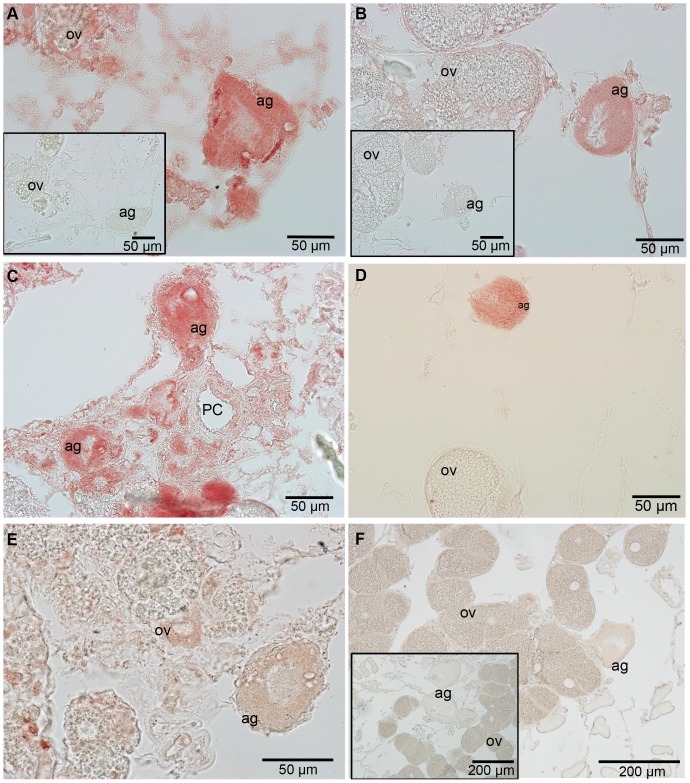
Polyclonal antibody for cp-52k adhesive protein in acorn and stalked barnacles. A, C & E) *A. improvisus* (acorn barnacle). B, D & F) *Lepas anatifera* (stalked barnacle). Insets are negative controls. Ag-adhesive gland, ov-ovarian tubules, PC-principal canal.

Polyclonal antibodies raised against adhesive proteins from *M. rosa* were examined to investigate whether they produced any localised cross-reactivity in the adhesive gland of *L. anatifera*. *A. improvisus* acted as a positive control and example of an acorn-type barnacle. The cp-52k antibody (ab-cp-52k) gave a positive reaction in the adhesive gland cells of both *A. improvisus* and *L. anatifera*, with the gland cells of replicate samples staining to varying degrees of red (Figure 5). Occasionally glands stained very faintly, leaving them hardly distinguishable from the negative control. Not only that, but the antibody was not entirely specific, with some cells within the ovarian tubules also showing a positive reaction in both *L. anatifera* and *A. improvisus*. Despite some positive reaction in the ovarian cells, it does appear that ab-cp-52k shows the presence of cp-52k in the adhesive glands of both *A. improvisus* and *L. anatifera*. Cp-52k appears to be homogenously spread throughout the cytoplasm of the adhesive gland and is not present in the nucleus. Sections of the canal system which transports glue to the outside of the barnacle body were present in one sample of *A. improvisus*, but this showed very little to no reaction to ab-cp-52k (see [23] for detailed description of adhesive glands).

**Figure 6:**
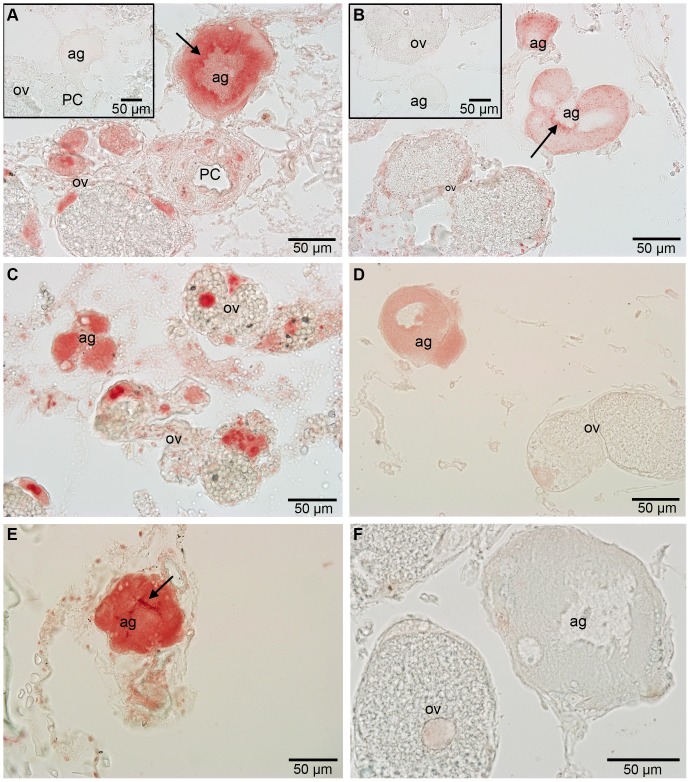
Polyclonal antibody for cp-68k adhesive protein in acorn and stalked barnacles. A, C & E) *A. improvisus* (acorn barnacle). B, D & F) *Lepas anatifera* (stalked barnacle). Insets are negative controls. Ag-adhesive gland ov-ovarian tubules PC-principal canal. Black arrows indicate intensely-stained areas.

Ab-cp-68k also gave a positive reaction in the adhesive gland cells, with the adhesive glands of *A. improvisus* being stained intensely red and the adhesive glands of *L. anatifera* having a more moderate reaction (Figure 6). Unlike ab-cp-52k, the reaction in the adhesive gland cells treated with ab-cp-68k was not always homogeneous, instead patches of intense colour were observed around the nucleus in some samples (Figure 6, black arrows). Ab-cp-68k was also not specific to the adhesive glands alone, with some ovarian cells staining quite intensely red in *A. improvisus* sections, a reaction that appeared to be localised in the nuclei of the ovarian cells. Again, the ovary in *L. anatifera* showed small patches of positive reaction. In a single *L. anatifera* sample out of four individuals investigated, there was no reaction to ab-cp-68k, for unknown reasons. Similar conclusions can be drawn from these results as from the ab-cp-52k results; ab-cp-68k is not entirely suitable for immunohistochemistry, yet the results do indicate that cp-68k is present in the adhesive gland cells of *A. improvisus* and *L. anatifera*, and possibly present in very small amounts in some cells surrounding the principal adhesive canal in *A. improvisus* (Figure 6A).

Immunohistochemistry with ab-cp-100k gave an unexpected result: the adhesive gland cells reacted very weakly to the antibody, while a strong reaction was seen in patches of the ovarian tubules of both *A. improvisus* and *L. anatifera* (Figure S2). Ab-cp-100k appears unsuitable for adhesive gland isolation in both positive control (*A. improvisus*) and *L. anatifera*.

### Sequence alignment

**Figure 7:**
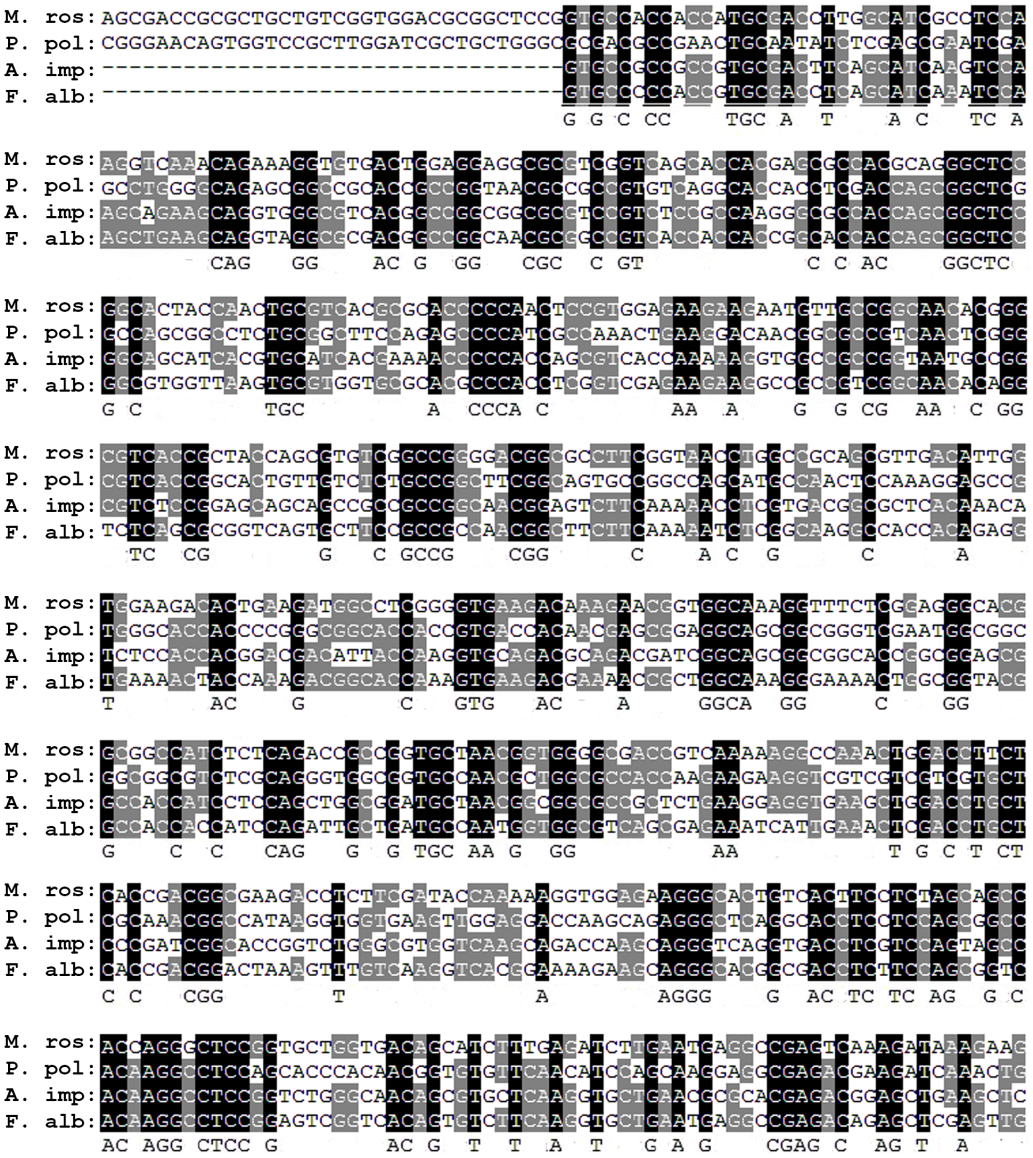
Extract from clustalW alignment of entire cp-19k genes from *M. rosa, A. improvisus* and *F. albicostatus* and EST sequences from *P. pollicipes*. Sequence conservation across four species is shown in black and across three species only is shown in grey. Dashed lines show base pairs that are not present in a sequence. Consensus sequence is included as the last line of each row.

Both AA sequences and cDNA sequences were aligned using ClustalW (MEGA5) and similarity between each taxon in the alignments was calculated using GeneDoc. The similarity for pairwise permutations of species is shown in Table 4. For example, cp-19k AA sequences were available for three species: *A. improvisus*, *F. albicostatus* and *M. rosa*. Gene alignment was performed with cDNA sequences from these species, along with a *P. pollicipes* EST sequence (Figure 7). Similarity between both AA sequences and cDNA sequences tended to decline as taxonomic distance increased. For instance, similarity between *A. improvisus* and *F. albicostatus* cDNA sequences was 49%; adding the *M. rosa* cDNA sequence resulted in similarity dropping to 36–40% and the addition of *P. pollicipes* ESTs resulted in similarity ranging from 26–36% (Table 4). Besides cp-19k, a range of similarities in a series of pairwise comparisons for other cement proteins/genes are given in Table 4 for different taxa.

**Table 4:** Similarity of barnacle adhesive cDNA and AA sequences between different species.

	cp-19k AA	cp-19k DNA	cp-20k AA	cp-20k DNA	cp-100k AA	cp-100k DNA
***A. improvisus, F. albicostatus***	60%	49%				
***A. improvisus, F. albicostatus, M. rosa***	42–44%	36–40%				
***A. improvisus, F. albicostatus, P. Pollicipes, M. rosa***		26–36%				
***F. albicostatus, A. amphitrite***			34–46%	29–36%	64%	49%
***F. albicostatus, A. amphitrite, M. rosa***			18–21%	19–30%	42–45%	37–40%
***F. albicostatus, A. amphitrite, P. pollicipes, M. rosa***						26–35%

Similarity was calculated using GeneDoc [41], based on Clustal W alignments created in Mega5 [40]. Similarity is calculated for species pairs, so for groups of more than two species a range of similarity is given. Two variants of cp-20k exist in *A. amphitrite* and both were included in alignments, resulting in a range of similarity values for the cp-20k two species comparison.

The barnacle adhesive protein cp-20k had sequences available from *M. rosa*, *F. albicostatus* and a pair of cp-20k variants from *A. amphitrite* [30]. Sequence conservation was low aside from repeating sequence motifs featuring Cys residues (Figure S3). Otherwise, the amino acid composition was quite disparate and, as seen in cp-19k, the pI differed between the three species; cp-20k had an alkaline pI in *F. albicostatus* (8.3) [28] and variant 1 from *A. amphitrite* (8.7) [30]; but acidic pI values were observed for this protein in *M. rosa* (4.7) [28] and *A. amphitrite* variant 2 (6.2) [30]. Simliarity between *A. amphitrite* (two variants) and *F. albicostatus* was calculated to be 34–46% (AA sequences) and 29–36% (cDNA sequences). Adding *M. rosa* to the alignments caused the similarity to drop to 18–21% (AA sequences) and 19–30% (cDNA sequences) (Table 4).

Amino acid sequences of cp-100k for *A. amphitrite*, *F. albicostatus* and *M. rosa* were easily aligned (not shown -see [31]). However, conservation between these sequences remained moderate (Table 4). Cp-100k cDNA sequences for the three aforementioned species were combined with *P. pollicipes* ESTs to create a cDNA alignment (not shown -see [31]); similarity between species ranged from 49% (*F. albicostatus* and *A. amphitrite*) to between 26 and 35% (all four species, taken two at a time) (Table 4).

PCR experiments were undertaken to isolate *L. anatifera* homologues of cp-19k, cp-20k and cp-100k using an extensive set of combinations of degenerate primers based on aligned sequences, however all attempts were unsuccessful (data not shown).

## Discussion

This study examines barnacle adhesive proteins across three taxonomic orders with the ultimate goal of identifying conserved adhesive domains in the entire group. We have focussed on lesser-known stalked barnacle adhesives in *Lepas anatifera* (order Lepadiformes) and *Pollicipes pollicipes* (order Scalpelliformes). Though similarity was low, the current research provides the first evidence of homologous cement proteins in distantly related barnacles: specifically positive immunostaining was observed in *L. anatifera* for 52 kDa and 68 kDa proteins from acorn barnacles (order Sessilia) and homologous adhesive genes (cp-19k and cp-100k) were identified in acorn barnacles and *P. pollicipes*.

SDS-PAGE indicated the range of adhesive proteins by mass in *L. anatifera*. The strongest and most consistent protein bands observed were 30, 90 and 110 kDa, with an additional strong band at 70 kDa observed more occasionally. These bands were also present in the closely-related species *Dosima fascicularis*. Protein masses observed in *L. anatifera* and *D. fascicularis* vary somewhat from what has been described in acorn barnacles; in acorn barnacles, small proteins of 19 and 20 kDa have been consistently observed in the adhesive and these are amongst the most ‘sticky’ proteins [27], [28], [46]–[48]. However protein bands below 30 kDa were faint and could not be consistently observed in repeated SDS-PAGE analysis in the present study.

A recent analysis of *Tetraclita japonica* found that cp-20k -a calcite binding protein [28] was absent from this acorn barnacle. This was suggested to be due to the fact that *T. japonica* possesses a membranous base [45]. Cp-20k may also be truly absent from other species, such as *L. anatifera* that possess a membranous base. However, the absence of bands of low molecular mass may also have been an experimental artefact of SDS-PAGE. Regarding the small sticky cp-19k protein, other results (see sequence alignment below) clearly indicated that *P. pollicipes*, which is a stalked barnacle, possesses this protein, although we do not yet know whether this is also the case in *L. anatifera*. The protein separation results indicated that some of the larger proteins, e.g., 68–70 kDa and 100–110 kDa, appear to be present in both acorn [34], [42], [43], [48] and stalked species (present study).

Fifty-two peptides were sequenced *de novo* from *L. anatifera* adhesive, some of them up to 16 amino acids in length. Examining these peptides revealed sequence conservation within lepadiform barnacles because three sequences M/YPALLVR, L/TVSLPR and FEDFLVN/SN(---)FSR were similar in *L. anatifera* and its relative *D. fascicularis*. However, the 52 peptides could not be matched to any databases, or to unpublished barnacle transcriptomes in *Amphibalanus amphitrite* and *T. japonica* [30], [44], [45] (pers. comm. Yue Him Wong, August 2013). The relatively short lengths of the peptide sequences and the taxonomic distance to acorn barnacles represented on databases may explain why no matches were found. Shared peptides between *L. anatifera* and *D. fascicularis* did not always originate from proteins of the same apparent mass (as indicated by protein separation gels) [42], [43].

A related observation was that identical *de novo* peptides appeared in proteins of different apparent masses in *L. anatifera*. In this case, the masses were usually (but not always) spaced regularly apart, for instance, identical peptide masses were found in each of the 70, 90 and 110 kDa bands (separated by ∼20 kDa). Naldrett [49] and Naldrett and Kaplan [46] reported regularly spaced protein bands in barnacle adhesives, which they suggested to indicate aggregates (dimers, etc.) of a single adhesive unit. Alternatively, this could be explained by certain bands containing more than one protein, such as a larger protein with the same smaller protein embedded within it. Multiple variants of adhesive proteins have been observed in another barnacle species (cp-20k, *A. amphitrite*, [30]) and in tubeworm and mussel adhesive proteins [22], [50], but it is unlikely that the shared peptides indicate protein variants in the present study, as the protein bands have significantly different masses.

Conservation of protein expression was examined by determining whether polyclonal antibodies raised in an acorn barnacle (*M. rosa*) displayed cross-reactivity to stalked barnacle (*L. anatifera*) adhesive glands. The acorn species *A. improvisus* was used as a positive control as this species contains the proteins that were targeted in this study. Antibodies against two of the three proteins tested, ap-cp-52k and ab-cp-68k, showed cross-reactivity in the adhesive glands of both *A. improvisus* and *L. anatifera*. The results therefore suggest homologous protein expression of cp-52k and cp-68k in both *Lepas anatifera* and acorn barnacles. The reactions were not entirely specific to the adhesive gland tissue and some sections of ovarian tissue stained positively. It is difficult to say whether this was because the antibodies in question were polyclonal or because they were generated from *M. rosa*. Immunohistochemistry is less specific when using polyclonal probes, however, lower specificity was considered to be a positive factor in the current case because polyclonal antibodies may counteract species-specific variations and allow a more broad examination across taxonomic groups [51], [52]. It is worth noting that some gland tissues only stained very faintly with ap-cp-52k and ab-cp-68k. This may indicate that protein synthesis is intermittent within adhesive gland cells (as noted previously by Kamino [53]). Western blotting would confirm whether these positive immunostaining results in *L. anatifera* correspond to any of the protein bands observed with SDS-PAGE.

The only stalked barnacle for which adhesive gene sequence information is available is *P. pollicipes*, due to the existence of published EST sequences as part of a taxonomic study [39]. That *P. pollicipes* possesses the cp-19k and cp-100k adhesive genes that are homologous to acorn barnacle genes has not been noted previously. *P. pollicipes* occupies an interesting taxonomic position because, although it is a stalked barnacle in the order Scalpelliformes, *P. pollicipes* is actually more closely related to the acorn barnacles than *Lepas*. The clade containing *Lepas* split approximately 35 million years prior to the split of the acorn/scalpelliform groups [54] (see Figure 2). Gene sequence alignments showed that sequence similarities between acorn barnacles and *P. pollicipes* were rather low and ranged from 26% to 36% (or 26–35%, depending on the gene – see Table 4). Within the acorn barnacles, sequence similarity tended to be higher, reaching up to 49% similarity in cDNA. Some genes were rather low in similarity irrespective of taxonomic closeness, such as cp-20k (19-36% cDNA similarity within the acorn barnacles). As noted above, this protein is absent in certain species [52] and occurs in variant forms [55]. By contrast, homologues of cp-100k have been found in more than seven acorn barnacle genera [8] and now in stalked barnacles as well (present study). Higher sequence similarity is apparent in the 100 kDa protein across the three species of acorn barnacles included in the current analysis (37–40% cDNA). Although this figure declined to 26-35% similarity once a stalked barnacle (*P. pollicipes*) was included, the picture which emerges is that some adhesive proteins (100 kDa) appear to be more conserved than others (20 kDa).

The significance of low sequence similarity, includes substantial changes to the pI of homologous cement proteins across species, which could have large affects on adhesion chemistry. For instance, cp-19k in *M. rosa* (5.8), is vastly different from the pI of the same protein in *A. improvisus* (10.3) and *F. albicostatus* (10.3) [27]. Added to this variability is the fact that none of the common post-translational modifications with adhesive attributes (e.g., DOPA or pSer) can be seen in the barnacle model. We suggest that an important step to understand the molecular basis for adhesion in barnacles is to identify conserved adhesive domains across all groups. Due consideration of taxonomic breadth can illustrate conserved domains, as seen in Table 1. Mussel adhesive protein fp-1 is conserved across a wide range of species within the family Mytilidae, but the primary sequences of all of the species could not be aligned until *Choromytilus chorus* was included; as only then was the relationship between the primary sequences and fp-1 repeated motif evident across the species (Table 1; [9], [56]–[60]).

Flexibility in protein primary structure may make the adhesive of barnacles highly adaptable to many different types of substrate; indeed *L. anatifera* has been observed attaching to surfaces with both high (metals, glass) and low surface energies (plastics including PET), as well as organic surfaces including algae, wood, feathers, mammalian fur, etc. By contrast, other species of barnacle do not foul man-made materials and are present only on intertidal rocks in very specific environmental conditions (e.g., [61]). This adaptability presents great challenges to understanding the mechanisms which cause barnacle adhesion and cohesion more generally. Even within ‘similar’ species of stalked barnacle, great differences are apparent; this study has shown that the adhesives of *L. anatifera* and *D. fascicularis* share similarities in protein mass and sequence structure, yet the adhesive of the first is a conventional adhesive plaque, while that of the second has become modified to become a buoyant ‘float’ [62]. Until more sequence data are available in a wider variety of taxonomic groups, identifying conserved adhesive domains and gaining insight into the relationship between sequence structure and protein function will remain elusive.

## Supporting Information

Figure S1 **SDS-PAGE adhesive protein separation in** ***Dosima fascicularis*** **.** SDS-PAGE adhesive protein separation in *Dosima fascicularis*. Prominent bands are indicated at 47, 63, 68, 85, 149, 205 kDa mass. Weaker and occasional bands of other masses are indicated in grey.
(TIF)

Figure S2 **Polyclonal antibody for cp-100k adhesive protein in acorn and stalked barnacles.** Polyclonal antibody for cp-100k adhesive protein in acorn and stalked barnacles. A, C, E & F) *A. improvisus* (acorn barnacle). B, D & G) *Lepas anatifera* (stalked barnacle). Insets show negative control. Ag-adhesive glands, ov-ovarian tubules, PC-principal canal.
(TIF)

Figure S3 **Alignment of entire cp-20k amino acid sequences from** ***A. amphitrite*** **(two variants),** ***F. albicostatus*** **and** ***M. rosa*** **.** Alignment of entire cp-20k amino acid sequences from *A. amphitrite* (two variants), *F. albicostatus* and *M. rosa*. Cys residues are aligned and highlighted in yellow. Identical residues found in all four proteins are highlighted in black; those conserved across only three are highlighted in grey. Dashed lines show residues that are not present in a sequence. Consensus sequence is included as the last line of each row.
(TIF)
