# Supplementary figures and images for: Adhesive Proteins of Stalked and Acorn Barnacles Display Homology with Low Sequence Similarities
Source: PLoS One. 2014 Oct 8;9(10):e108902. doi: 10.1371/journal.pone.0108902 (PMC4189950; doi:10.1371/journal.pone.0108902)

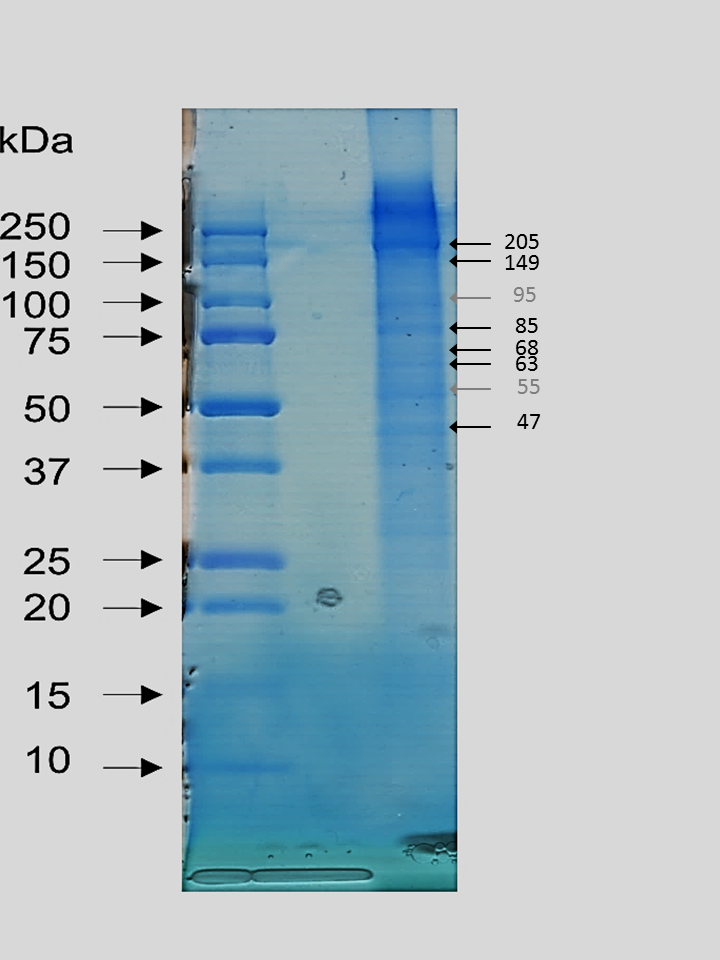

Supplement: Figure S1 — SDS-PAGE adhesive protein separation in Dosima fascicularis . SDS-PAGE adhesive protein separation in Dosima fascicularis. Prominent bands are indicated at 47, 63, 68, 85, 149, 205 kDa mass. Weaker and occasional bands of other masses are indicated in grey. (TIF) [file pone.0108902.s001.tif]

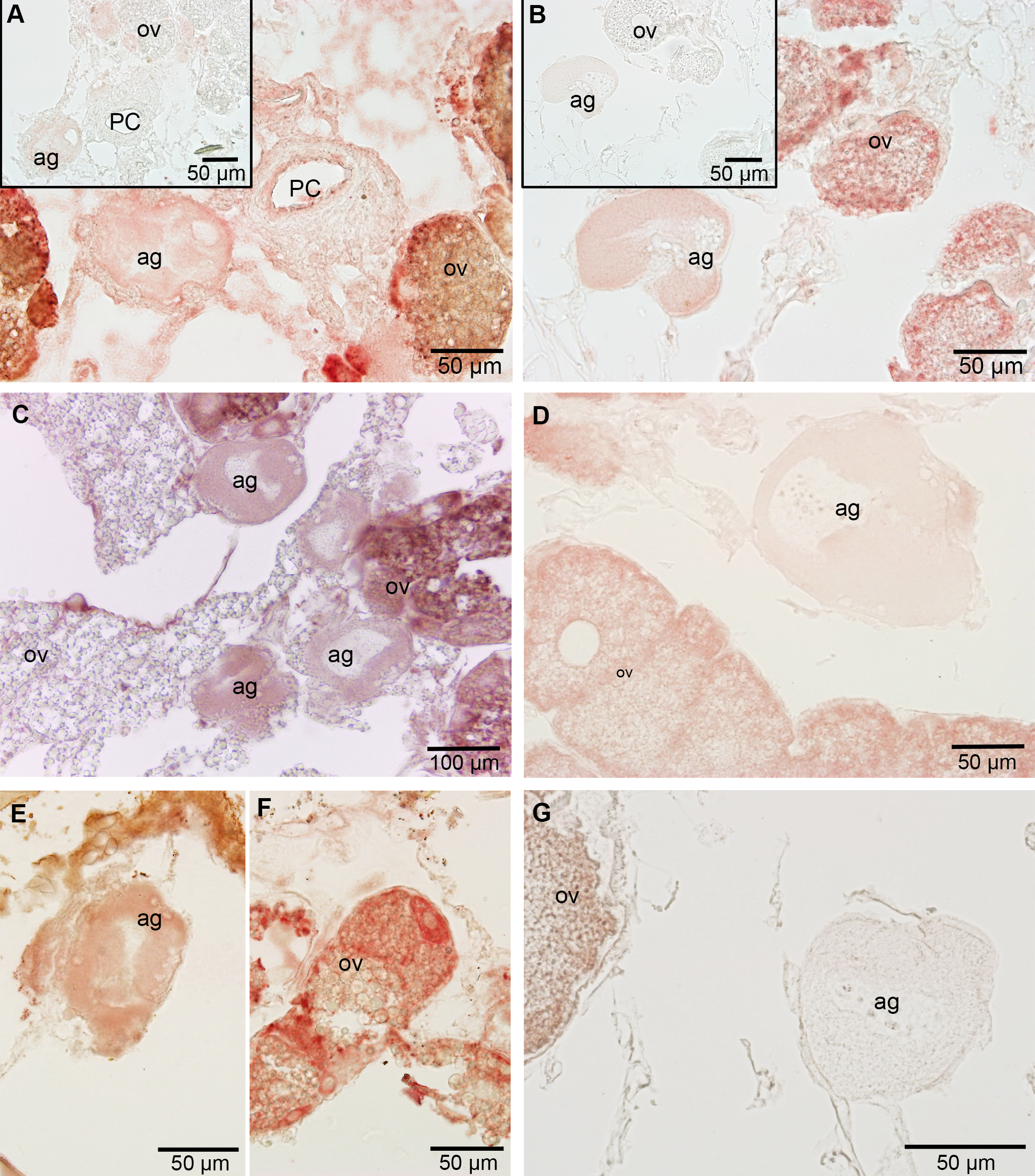

Supplement: Figure S2 — Polyclonal antibody for cp-100k adhesive protein in acorn and stalked barnacles. Polyclonal antibody for cp-100k adhesive protein in acorn and stalked barnacles. A, C, E & F) A. improvisus (acorn barnacle). B, D & G) Lepas anatifera (stalked barnacle). Insets show negative control. Ag-adhesive glands, ov-ovarian tubules, PC-principal canal. (TIF) [file pone.0108902.s002.tif]

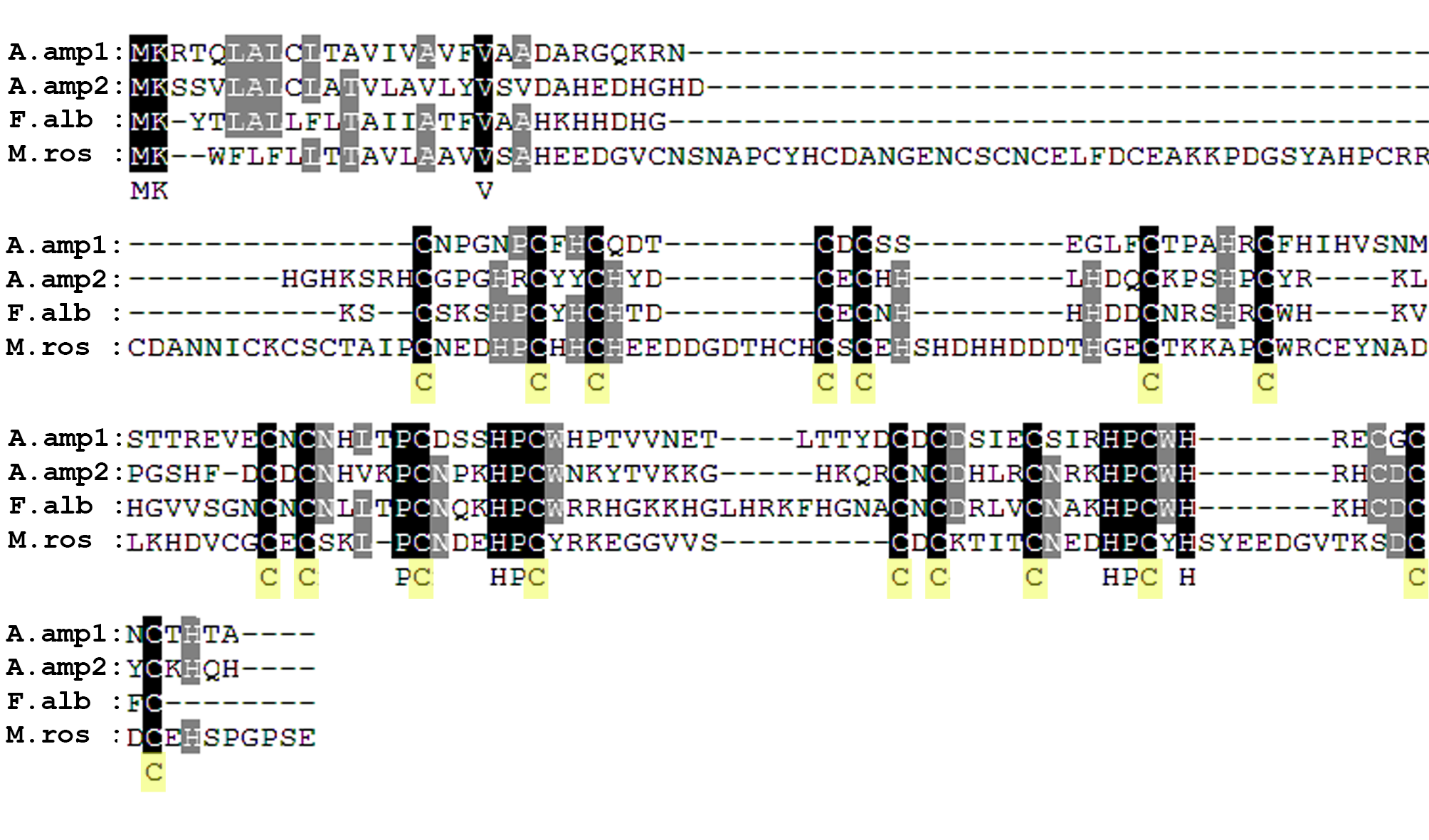

Supplement: Figure S3 — Alignment of entire cp-20k amino acid sequences from A. amphitrite (two variants), F. albicostatus and M. rosa . Alignment of entire cp-20k amino acid sequences from A. amphitrite (two variants), F. albicostatus and M. rosa. Cys residues are aligned and highlighted in yellow. Identical residues found in all four proteins are highlighted in black; those conserved across only three are highlighted in grey. Dashed lines show residues that are not present in a sequence. Consensus sequence is included as the last line of each row. (TIF) [file pone.0108902.s003.tif]
